# From Surviving to Thriving: Overcoming Early Career Challenges in Transplant Infectious Diseases

**DOI:** 10.1111/tid.70281

**Published:** 2026-07-24

**Authors:** Courtney E. Harris, Chelsea A. Gorsline, Ruth Adekunle, Christina Vojtek, Eric Altneu, Julia Bini Viotti, Michael Scolarici, Chia‐Yu Chiu, Rebecca N. Kumar

**Affiliations:** ^1^ Division of Infectious Diseases Medical University of South Carolina Charleston South Carolina USA; ^2^ Division of Infectious Diseases University of Kansas Medical Center Kansas City Kansas USA; ^3^ Division of Infectious Diseases Indiana University Indianapolis Indiana USA; ^4^ Division of Infectious Diseases Temple University Philadelphia Pennsylvania USA; ^5^ Division of Infectious Diseases Miami Transplant Institute University of Miami Miami Florida USA; ^6^ Division of Infectious Diseases University of Wisconsin–Madison Madison Wisconsin USA; ^7^ Division of Infectious Diseases Department of Medicine University of Colorado School of Medicine Aurora Colorado USA; ^8^ Division of Infectious Disease and Tropical Medicine Georgetown University Washington, DC USA

**Keywords:** career development, early career, mentorship

## Abstract

Transplant infectious diseases (TID) is a highly specialized subspecialty situated at the intersection of immunosuppression, emerging pathogens, antimicrobial stewardship, and multidisciplinary transplant care. Early‐career TID physicians must balance high‐acuity clinical responsibilities with expectations for scholarly productivity, programmatic engagement, and leadership development while establishing credibility within transplant programs. This “Experience from the Field” report highlights key domains that shape early‐career success in TID, including mentorship, trainee development, skill acquisition, research strategy, time management within unpredictable clinical environments, maintenance of scientific currency, professional visibility, and long‐term sustainability. Intentional alignment of clinical, academic, and personal priorities is required to move from professional survival toward sustained impact within the evolving landscape of TID.

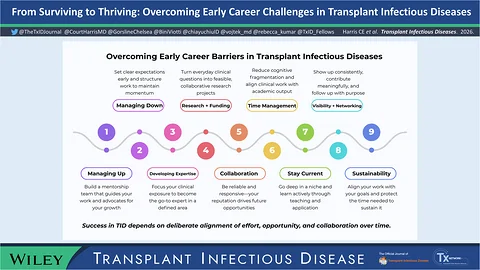

## Graphical abstract

Practical strategies to overcome early‐career challenges in transplant infectious diseases are presented, emphasizing mentorship, focused expertise, collaboration, and sustainability. Long‐term success depends on aligning effort, opportunity, and collaboration over time.

**Abbreviations**
AST IDCOP: American Society of Transplantation Infectious Diseases Community of Practice
FTE: full‐time equivalent
TID: transplant infectious disease

## 1 Introduction

The field of transplant infectious diseases (TID) occupies a distinct position within transplantation. The field requires the integration of opportunistic infection risk assessment, antimicrobial stewardship in immunocompromised hosts, evaluation of donor‐derived infections, and interpretation of increasingly complex diagnostics, including molecular and metagenomic platforms [1]. Clinical decision‐making frequently occurs within multidisciplinary transplant ecosystems involving surgeons, transplant physicians, pharmacists, and coordinators, often under time‐sensitive conditions.

For early‐career physicians, these clinical demands coincide with expectations for scholarly productivity, quality improvement, and institutional engagement. However, there is no universal approach to handling this as a junior faculty member, as transplant programs vary widely in structure, protected time, and research infrastructure. As a result, early‐career development in TID is often less linear than in other subspecialties and requires deliberate navigation of competing priorities. Recent discussions around the TID workforce have emphasized the need for structured mentorship, educational resources, and career development pathways to support sustainability in the field [2, 3, 4, 5].

To better understand and contextualize the challenges faced by early‐career TID attendings, the Transplant Infectious Diseases Early Career Network (TxID ECN) convened an in‐person breakfast session, “Breakfast Bites & Barriers: A Peer Discussion of How to Overcome Hurdles in the Early Career Stage in Transplantation,” at the World Transplant Congress 2025 in San Francisco, USA. The session included small‐group discussions with a lead moderator (C.E.H.) and designated table moderators, all of whom were TID physicians (R.A., C.V., E.A., J.B.V., M.S., C.C., and R.N.K.) who guided discussion and documented key insights. While attendance varied throughout the session, approximately 80–100 participants were present, with strong representation from the TID field, as well as participation from other disciplines, including transplant medicine physicians, surgeons, pharmacists, advanced practice providers, and trainees across geographic regions. Discussions focused on practical aspects of early‐career development, with the specific discussion questions provided in Appendix A.

## 2 Mentorship Within a Multidisciplinary Environment

Mentorship in TID is inherently multidimensional. Unlike more siloed subspecialties, TID operates across clinical, research, and administrative domains that often span divisions. A single mentor is rarely able to provide guidance across all aspects of early career development.

Clinical mentorship is particularly important in TID, where nuanced decision‐making is required for complex scenarios such as CMV resistance, invasive fungal infections, or donor‐derived infection risk. These decisions are frequently made in real time and within multidisciplinary settings, reinforcing the importance of mentorship that extends beyond traditional academic advising. Clinical mentorship could be provided by a more senior TID physician at the institution, or, if this is not available, may need to come from mentors at other institutions. Access to mentors and near‐peer colleagues for case discussion is particularly valuable in complex, guideline‐limited scenarios where published evidence is limited [6].

Complementarily, research mentorship supports the development of study design, data interpretation, and integration into multicenter collaborations, which are increasingly central to TID scholarship. Research mentors can often be identified through ongoing institutional projects, clinical and translational research programs, or by engaging with investigators leading studies aligned with one's clinical interests. For example, the American Society of Transplantation Infectious Diseases Community of Practice (AST IDCOP) offers multiple subgroups that connect clinicians with shared areas of interest and foster participation in multicenter studies [7].

Sponsorship within transplant programs represents an additional, and often underrecognized, component of mentorship. Sponsors advocate for protected time, inclusion in transplant selection committees, and visibility within institutional structures, all of which can significantly influence early‐career trajectory. Partnering with the leadership of solid organ transplant (SOT), cellular therapies, or mechanical circulatory support programs can promote integration into programmatic leadership, foster collaborative opportunities, and support long‐term career advancement within transplant systems.

Collectively, clinical mentors, research mentors, and sponsors create the foundation of a mentorship network, which often evolves over time. As clinical and academic interests become more defined, external mentorship through national societies, collaborative research networks, or multicenter studies may be necessary to support niche areas of expertise. This is particularly relevant in TID, where areas such as virology, fungal infections, or donor‐derived infection surveillance may require mentorship beyond local institutional expertise. In practice, this involves building a mentorship team rather than relying on one single mentor to encompass clinical, research, and educational goals. Taking care to approach meetings with clear questions or agendas to maximize actionable feedback and provide regular updates so mentors remain engaged and positioned to advocate for opportunities as they arise. These approaches position mentorship not only as a source of guidance but as a critical driver of opportunity and career progression in TID.

## 3 Mentoring the Next Generation

In addition to being mentees, early‐career TID physicians are also tasked with mentoring the next generation of learners. Effective mentorship benefits from setting expectations early and making the process explicit. Clarifying a trainee's goals at the outset—whether clinical exposure, authorship, or skill development—allows for alignment of responsibilities.

From a clinical standpoint, early‐career TID physicians frequently supervise medical students, residents, and fellows rotating on transplant services. Effective clinical mentorship requires deliberate structure and adaptation to trainee experience [8]. For learners new to transplantation, focused teaching around infection timelines, prophylaxis strategies, and the clinical implications of immunosuppression provides a necessary framework. For more advanced trainees, the emphasis shifts toward refining diagnostic reasoning in complex scenarios, including evaluation of breakthrough infections, interpretation of discordant microbiologic data, management of multidrug‐resistant organisms, and donor evaluation. Accordingly, effective mentorship requires early assessment of each trainee's prior exposure and skill level to appropriately tailor TID teaching and clinical responsibilities.

When involving trainees in scholarly work, outlining the structure of a manuscript or project before writing begins, assigning discrete sections, and establishing firm interim deadlines can prevent projects from stalling. Reviewing work iteratively rather than waiting for a full draft often improves both efficiency and learning.

Providing graduated autonomy is also critical in both research and clinical settings. Mentorship is most effective when aligned with the trainee's next step, with deliberate focus on building skills that directly support their progression. This may include guiding a medical student through a differential diagnosis for fever after a heart transplant, working with a resident on structuring and writing a manuscript, or supporting a fellow in developing a grant proposal. Allowing trainees to take initial ownership of data gathering, literature review, or draft writing, while maintaining regular check‐ins, promotes independence without a loss of direction. At the same time, being appropriately hands‐on ensures that expectations are clear and standards are maintained. Small, practical habits, such as reviewing one section at a time or setting short turnaround timelines, can meaningfully improve momentum.

Mentorship in TID also extends beyond individual projects. Involving trainees in transplant selection discussions, stewardship decisions, or donor evaluation conversations can provide insight into the broader context of TID practice. These experiences not only enhance clinical understanding but also help trainees appreciate the complexity and collaborative nature of the field. Taken together, a structured, goal‐oriented, and hands‐on approach to mentorship supports both trainee development and scholarly productivity, while reinforcing the pipeline of future TID physicians.

## 4 Developing Expertise and Academic Identity

The breadth of TID can make it difficult for early‐career physicians to establish a focused area of expertise [9]. Clinical responsibilities span pretransplant candidate evaluation, donor evaluation, inpatient consultative care, and longitudinal outpatient management, often within the same week or even day. At the same time, opportunities for academic engagement are abundant, including stewardship initiatives, transplant protocols, collaborative research projects, selection committees, and morbidity/mortality conferences. Without intentional focus, effort can become fragmented across too many domains without meaningful depth in any one area.

Early‐career physicians frequently feel pressure to excel simultaneously across clinical care, education, research, and administrative leadership. While broad engagement is often encouraged, long‐term career advancement is more commonly achieved by developing a primary area of strength supported by one or two complementary domains. Establishing this professional identity early can help prioritize opportunities, guide allocation of effort, and reduce the risk of overextension.

Developing expertise in TID is most effective when anchored in clinical practice. Identifying patterns in one's consult service, such as frequent involvement in CMV management, invasive fungal infections, or antimicrobial stewardship challenges, can help define an initial area of focus. This focus can also be shaped by one's role within the transplant program, including consistent participation in organ‐specific selection conferences or clinical services. For example, physicians aligned with heart or lung programs may develop expertise through regular involvement in thoracic organ selection and perioperative care, while others may build a focus in hematopoietic cell transplantation or cellular therapies through dedicated clinical exposure in those areas. Leaning into these recurring clinical problems allows for simultaneous growth in clinical expertise and generation of research questions that are both relevant and feasible. Reviewing cases in real time, maintaining a running list of clinical questions, and revisiting complex cases after resolution can help consolidate learning and identify areas for deeper investigation [10].

Establishing an academic identity requires translating clinical exposure into scholarly output. This often begins with pragmatic projects, such as case series, retrospective cohort studies, quality improvement initiatives, or protocol development that are closely aligned with clinical work. Starting with projects that can be completed within existing clinical workflows increases the likelihood of early productivity and helps build momentum. Over time, these efforts can evolve into more complex investigations, including multicenter collaborations or interventional studies.

Intentional alignment of activities is critical. These early‐career decisions, including prioritization of opportunities, negotiation of roles, and alignment with long‐term goals, have been highlighted as key determinants of early‐career success in TID [9]. Transplant programs frequently present numerous opportunities for committee participation, protocol development, and interdisciplinary initiatives. While these roles are valuable, selectively engaging in those that align with a defined clinical or research focus allows for deeper contribution and recognition. In practice, this may involve prioritizing projects that intersect with one's area of interest, declining those that do not, and revisiting commitments periodically to ensure continued alignment.

Skill development in TID is iterative and grounded in clinical experience. Growth often involves deepening understanding of transplant‐specific pharmacology, refining interpretation of complex diagnostics, and developing comfort with clinical uncertainty. Case‐based discussions, multidisciplinary collaborations, and longitudinal follow‐up of patients remain central to this process.

Ultimately, academic identity in TID is built on clinical credibility. Establishing a clear area of focus allows early‐career physicians to contribute meaningfully within transplant programs and creates a foundation for subsequent scholarly work.

## 5 Research Strategy and Funding Pathways

Developing a research program in TID is most effective when grounded in a defined clinical focus and derived from routine patient care. Participation as a co‐investigator on ongoing studies, including multicenter cohorts or clinical trials, provides exposure to study design, data coordination, and regulatory processes. In a field characterized by relatively small patient populations and complex clinical questions, collaboration is often necessary to generate meaningful data. Reliability, responsiveness, and the ability to move projects forward are key factors that influence continued inclusion in collaborative efforts.

Funding pathways in TID are diverse and often interdisciplinary. In addition to traditional infectious diseases funding mechanisms, early‐career investigators may benefit from pursuing foundation grants, institutional pilot funding, or opportunities through organ‐specific, transplant, or pathogen‐focused societies. For research questions that intersect multiple disciplines, exploring funding mechanisms outside of infectious diseases can broaden funding opportunities and increase competitiveness.

For individuals pursuing a research‐intensive career, the transition from participating in research projects to serving as an independently funded investigator represents a distinct career milestone. Developing relationships with experienced investigators who have successfully obtained extramural funding can provide valuable guidance regarding grant development, protected time, team science, and long‐term career planning. For those pursuing federal funding pathways, mentorship from investigators with a track record of NIH‐funded research may be particularly valuable.

## 6 Time Management in a High‐Acuity Clinical Field

Time management in TID is uniquely challenging due to the unpredictability and intensity of clinical care. Unlike other academic settings, clinical responsibilities are not easily contained within scheduled blocks. Donor evaluations, curbside consultations, program coordinator questions, and urgent consultative needs frequently interrupt planned work, making it difficult to protect time for scholarship. This tension between clinical demands and academic productivity is well described across academic medicine and contributes to early‐career burnout and attrition if not addressed deliberately [11, 12].

In practice, effective time management in TID requires both structural and behavioral strategies. Structurally, delineating dedicated time for research, administrative work, or writing, even if imperfectly protected, helps create cognitive separation between clinical and academic tasks. Assigning general themes to days or half‐days, such as research‐focused or clinic‐focused time, can provide a framework that allows work to progress despite interruptions. At the same time, accepting that these boundaries will occasionally be disrupted is important to maintaining flexibility within a high‐acuity field.

Equally important is developing habits that preserve time within the clinical day. Completing documentation in real time or by the end of the day prevents the accumulation of work that encroaches on academic time. Not every communication requires immediate response, and triaging messages based on urgency can reduce the fragmentation of attention [13, 14]. Small adjustments in workflow, such as batching similar tasks or setting brief, focused writing intervals, can meaningfully improve productivity even during busy service weeks.

Protecting time also requires intentional alignment of commitments. Early‐career TID physicians are frequently asked to participate in organ selection committees, often requiring several hours of multidisciplinary discussion for a single organ group, with most institutions supporting multiple transplant programs, as well as transplant initiatives and research projects. While many of these opportunities are worthwhile, not all are feasible, particularly in the absence of dedicated full‐time equivalent (FTE) support. Evaluating these commitments in the context of long‐term career goals and current bandwidth is essential. Projects that are closely aligned with one's developing area of expertise and have a clear path to completion are more likely to result in meaningful output.

Finally, communication with leadership is critical. Expectations for clinical effort, academic productivity, and programmatic involvement must be clearly defined. Providing objective data on clinical workload and scholarly output can support discussions around protected time and resource allocation. In TID, where clinical demand is unlikely to decrease, sustainable productivity depends not on working more, but on working with intentional structure and clear prioritization.

## 7 Maintaining Scientific Currency

Maintaining scientific currency in TID requires a deliberate and structured approach. In practice, staying current is most sustainable when integrated into routine workflow. Regularly reviewing journal tables of contents, setting targeted alerts for key journals, and focusing on a defined subset of topics aligned with one's clinical and academic interests help prioritize high‐yield reading. Developing depth within a focused area allows for more meaningful interpretation and application of new evidence, rather than superficial familiarity across multiple domains.

Active engagement with the literature further reinforces learning. Preparing conference presentations, leading case‐based discussions, or writing review articles requires a deeper evaluation of available data and improves retention. Participation in multidisciplinary transplant conferences also provides exposure to evolving practices across specialties and highlights clinically relevant questions that may not yet be well represented in the literature.

Prioritizing studies by reviewing abstracts or summaries can identify studies that warrant deeper review, while full‐text reading can be reserved for work that directly informs clinical decision‐making or aligns with one's area of focus. Digital tools, including curated journal alerts and emerging summarization platforms using artificial intelligence, may support this process, though verification of primary sources remains critical.

Maintaining scientific currency has been associated with improved clinical decision‐making and patient outcomes [15]. However, sustainability depends on consistency rather than volume. Integrating literature review into existing clinical and academic activities, rather than approaching it as a separate task, allows for continued engagement without overwhelming competing priorities.

## 8 Visibility and Professional Network Development

Visibility within TID is closely tied to collaboration, and deliberate effort is often required to build a professional network in a relatively small and interdisciplinary field. Establishing visibility early in one's career can facilitate access to research opportunities, mentorship, and leadership roles.

In practice, visibility is most effectively built through consistent participation in environments where transplant decisions and scholarship occur. Presenting at institutional conferences across disciplines, such as transplant surgery or organ‐specific medicine subspecialty forums, broadens recognition beyond infectious diseases and reinforces one's role within the transplant program. These settings also provide opportunities to identify shared clinical challenges that may serve as the basis for collaborative work.

National and international meetings remain important for expanding networks beyond one's institution. Approaching these meetings with specific goals, such as identifying individuals working in similar areas or following up on recent publications, can make networking more intentional and productive. Brief, focused introductions that reference a shared interest or a specific project are often more effective than general conversation. Following initial interactions with a timely and purposeful follow‐up, such as sharing an idea for collaboration or continuing a discussion, helps convert introductions into ongoing professional relationships. The AST IDCOP represents one example of a structured forum for engagement through topic‐focused workgroups, many of which are conducted virtually and have lower barriers to participation. Similar opportunities exist through other professional societies and multidisciplinary groups (e.g., ISHLT, ASTS), offering additional platforms for collaboration and sustained engagement.

Visibility is also reinforced through scholarly output. Presentations, publications, and participation in multicenter projects increase recognition within the field and often lead to additional opportunities for collaboration. In a small subspecialty such as TID, reputation is frequently built on reliability and the ability to contribute meaningfully to collaborative efforts. Responding promptly, meeting deadlines, and maintaining clear communication are practical behaviors that influence whether individuals are invited to participate in future work.

Digital platforms may further extend visibility, though their use should be aligned with individual preferences and professional goals. Thoughtful engagement, such as sharing relevant work or contributing to field‐specific discussions, can complement traditional academic dissemination. Ultimately, building a professional network in TID is less about isolated interactions and more about sustained engagement. Consistent participation, follow‐through, and alignment with one's developing area of expertise are central to establishing a durable presence within the field.

## 9 Sustainability and Work–Life Integration

Sustaining a career in TID requires deliberate attention to workload, boundaries, and alignment of professional responsibilities with personal priorities. The demands of the field, including high‐acuity clinical care, unpredictable schedules, and expectations for academic productivity, can make balance difficult to achieve and maintain. These demands are further compounded by TID‐specific responsibilities, such as overnight donor call coverage, organ selection responsibilities, frequent teaching on immunocompromised hosts to multiple levels of trainees, and transplant‐specific protocol development. Efforts to improve well‐being in physicians have consistently demonstrated that burnout is closely linked to workload, inefficiency, and lack of control over one's time [16].

In practice, sustainability often depends on recognizing that balance is not static. Periods of increased professional demand, such as preparing a grant, managing heavy clinical service, or completing major academic projects, may temporarily shift priorities toward work. Planning for recovery time following these periods and intentionally re‐engaging in non‐work activities is critical to maintaining long‐term balance.

Establishing boundaries is equally important. Defining limits around availability, whether related to after‐hours work or clinical responsibilities, and maintaining those boundaries helps prevent the gradual expansion of workload over time. Not every request or opportunity requires immediate acceptance, and developing a consistent approach to declining or deferring commitments is essential to preserving time and energy for high‐priority work. Suggesting colleagues who may be a good fit for an opportunity one declines can also help to build relationships while saying no. In TID practice, where curbside consultations are common, intentionally converting appropriate discussions into formal consults represents one strategy to align clinical effort with recognition and compensation.

Sustainability is also supported by alignment. In TID, prioritizing work that aligns with one's transplant focus—whether through organ‐specific clinical care, antimicrobial stewardship in immunocompromised hosts, or protocol development—can improve both productivity and satisfaction. In contrast, accumulating responsibilities that are misaligned with long‐term objectives increases the risk of burnout without contributing to meaningful career growth.

Finally, collaboration plays an important role in maintaining balance. Sharing responsibility across teams, involving trainees in appropriate aspects of clinical and scholarly work, and avoiding isolated approaches to projects can reduce individual burden while enhancing productivity. In practice, this includes leveraging the expertise of transplant coordinators to facilitate communication and coordinate laboratory monitoring, transplant pharmacists to guide antimicrobial selection, dosing, and drug–drug interactions, and other multidisciplinary team members who support the complex care of immunocompromised patients. In a field as multidisciplinary as TID, leveraging the strengths of the broader team is both practical and necessary. Ultimately, work–life integration in TID is not achieved through perfect balance, but through intentional prioritization, boundary setting, and periodic recalibration over time. These themes are summarized as high‐yield, actionable strategies for early career development in TID (Table 1).

**TABLE 1:** High‐yield strategies for early career development in transplant infectious diseases.

Domain	Common challenge	High‐impact strategies
Mentorship (managing up)	Limited access to mentors who understand TID complexity	Build a mentorship team with distinct roles (clinical, research, and sponsorship) Come to meetings with specific questions or agendas Provide regular updates to maintain engagement and facilitate advocacy
Mentorship (managing down)	Variable trainee experience and project stagnation	Define trainee goals early and align expectations Outline projects before writing begins Set firm interim deadlines and review work iteratively
Developing expertise	Difficulty narrowing focus in a broad field	Align with a transplant program or organ system and attend selection conferences consistently Identify recurring consult patterns and build depth in those areas Maintain a running list of clinical questions to guide learning and research
Research and funding	Unclear entry point and limited early funding	Start with pragmatic, clinically derived projects feasible within current workflow Participate as a reliable co‐investigator on ongoing studies Pursue diverse funding sources, including institutional and cross‐disciplinary opportunities Seek mentors with experience obtaining extramural funding aligned with your career goals
Collaboration	Silos across specialties and limited access to multicenter work	Engage in multidisciplinary meetings and propose research questions in real time Follow up promptly with potential collaborators Build a reputation through reliability and responsiveness
Time management	Clinical demands encroaching on academic productivity	Block dedicated time for writing or research and treat it as non‐negotiable Complete documentation in real time to prevent backlog, and for any task that takes < 10–15 min, finish it immediately Use short, focused work intervals to maintain progress Batch clinical decision‐making to avoid cognitive switching multiple times per day Standardize repetitive cognitive tasks using pretransplant evaluation templates, vaccine checklists, and dot phrases for common clinical diagnoses
Maintaining scientific currency	Overwhelming volume of literature	Focus on a defined clinical or research niche Use journal alerts and table‐of‐contents review to triage reading Reinforce learning through teaching or presentations rather than passive reading
Visibility and networking	Limited recognition outside immediate team	Present across transplant disciplines, not just infectious diseases Approach meetings with specific goals and follow up with purpose Build visibility through reliability and meaningful contributions
Sustainability	Risk of burnout and misaligned commitments	Align activities with long‐term career goals Establish and maintain boundaries around time and availability Plan recovery after high‐intensity periods

## 10 Conclusion

Early‐career development in TID requires deliberate navigation across multiple interconnected domains, including mentorship, trainee development, expertise acquisition, research engagement, time management, maintaining scientific currency, professional visibility, and long‐term sustainability. Success within this field is not defined by mastery of any single domain, but by the ability to align these elements in a manner that supports both professional growth and personal longevity. Collectively, these strategies support a transition from early career uncertainty to a more deliberate and sustainable trajectory. In TID, long‐term success is defined not by maximizing every opportunity, but by deliberate investment in the work that best aligns with one's professional identity, collaborators, and long‐term goals.

## Author Contributions

**Eric Altneu**: writing – review and editing, data curation, writing – original draft. **Chelsea A. Gorsline**: conceptualization, writing – review and editing, writing – original draft. **Christina Vojtek**: data curation, writing – review and editing, writing – original draft. **Michael Scolarici**: writing – original draft, writing – review and editing, data curation. **Ruth Adekunle**: writing – review and editing, data curation, writing – original draft. **Courtney E. Harris**: conceptualization, writing – original draft, writing – review and editing, visualization, investigation, supervision. **Chia‐Yu Chiu**: writing – original draft, writing – review and editing, data curation. **Rebecca N. Kumar**: writing – original draft, writing – review and editing, conceptualization, data curation. **Julia Bini Viotti**: writing – review and editing, data curation, writing – original draft.

## Conflicts of Interest

The authors declare no conflicts of interest.

## Supporting information




**Supplementary**: Visual Abstract

## Appendix A Question prompts for moderators at World Transplant Congress Early Career Session

### A.1

Question #1: ***MANAGING UP (MENTORS)***

How do you find and nurture relationships with mentors?

How do you know when to end a relationship or seek new counsel?

How do you know when you need an external mentor (i.e., to fill knowledge/expertise gaps)?

If you found mentors outside your department or your institution, how did you go about doing this?

Question #2: ***MANAGING DOWN (MENTEES)***

How do you encourage and support junior trainees/medical students as someone also navigating their own professional development?

What are successful strategies you've enacted to improve relationships and project management with mentees?

Question #3: ***ACQUIRING SKILLS***

What key skills should early career academics focus on developing to succeed in transplantation research and practice?

Are there any specific conferences, online tools, or other resources you have found helpful?

How can early career researchers develop and refine their presentation skills for conferences and seminars?

Question #4: ***RESEARCH (FUNDING + COLLABORATIONS)***

What are the most effective strategies for securing research funding and grants early in your career? What steps can you take to find and engage in interdisciplinary research collaborations?

Question #5: ***TIME MANAGEMENT***

How can early career academics effectively manage their time and prioritize tasks?

What strategies have you used that have maximized your productivity at work?

Question #6: ***STAYING UP TO DATE***

How can early career academics ensure they stay current with the latest advancements and innovations in transplantation research and technologies?

What resources do you regularly reach for to stay up to date?

Question #7: ***RECOGNITION OF WORK & NETWORKING***

What specific actions can early career academics take to build a strong online presence and reputation in their field?

What actionable steps can be taken to increase the visibility and impact of one's research within the transplantation field?

What other practical steps can early career academics take to build a robust professional network within the transplantation community?

Question #8: ***WORK‐LIFE BALANCE***

What are practical ways to maintain a healthy work‐life balance while pursuing demanding academic goals?

What do you do when you are not achieving the balance you want and how do you decide how to say no to certain opportunities?
